# Photoacoustic tomography of fingerprint and underlying vasculature for improved biometric identification

**DOI:** 10.1038/s41598-021-97011-1

**Published:** 2021-09-02

**Authors:** Wenhan Zheng, Diana Lee, Jun Xia

**Affiliations:** Optical & Ultrasonic Imaging Laboratory, Department of Biomedical Engineering, University at Buffalo, The State University of New York, Buffalo, NY 14260 USA

**Keywords:** Optical imaging, Ultrasound

## Abstract

Capitalizing on the photoacoustic effect, we developed a new fingerprint sensing system that can reveal both fingerprints and underlying vascular structures at a high spatial resolution. Our system is built on a 15 MHz linear transducer array, a research ultrasound system, and a 532-nm pulsed laser. A 3D image was obtained by scanning the linear array over the fingertip. The acquired fingerprint images strongly agreed with the images acquired from ultrasound. Additional experiments were also conducted to investigate the effect of acoustic coupling. We discovered that high-quality fingerprint and vessel images can be acquired from both wet and dry fingers using our photoacoustic system. The reduced subdermal features in dry coupling can be enhanced through post-processing. Compared to existing fingerprint scanners, the photoacoustic approach provides a higher quality 3D image of the fingerprint, as well as unique subdermal vasculature structures, making the system almost impossible to counterfeit.

## Introduction

Biometric sensing is being used more frequently to identify authentication in consumer devices, and also in banks and military bases. Biometric features can include iris patterns, facial features, palm prints, and fingerprints. Among these, fingerprints are the most widely used, due to their low misrecognition rate and user-friendly pattern^1,2^. Existing fingerprint sensors can be categorized into three different types based on their operating principle: optical, capacitive, and ultrasonic. Optical sensors capture the high-resolution optical contrasts between ridges and valleys of the fingerprint. Optical sensors are widely used in commercial and governmental settings^3^. Capacitive sensors measure the capacitance difference between ridges and valleys, and are the most common type of sensor used in smartphones^4^. Ultrasonic sensors utilize pulse-echo ultrasound to measure the impedance mismatch between ridge and valley. Recent advances in under-screen fingerprint sensors are mainly based on ultrasound^5^.

Although these imaging modalities provide high-resolution fingerprint images, the fingerprint information is represented in 2D space. While ultrasonic sensor could provide depth information, it has limited sensitivity to subdermal vascular structures. Therefore, these systems are vulnerable to attacks via spoof fingerprints^6^. To resolve this issue, researchers have proposed various anti-spoofing techniques. A survey by Marasco et al.^7^ summarized different types of spoof attacks and classified counter-measurement approaches as either hardware- or software-based. Hardware-based methods involve several vitality features such as temperature, electrical conductivity, and skin resistance. These features are measured and combined with fingerprint images to improve recognition accuracy. However, these approaches require additional sensing modalities, making the combined system complex and expensive. Software-based approaches utilize data from the existing fingerprint system and do not require additional hardware. Post-processing algorithms are developed to distinguish artificial fingerprints. However, most of these algorithms are learning-based and require a large amount of training data. The performance and accuracy of these algorithms are highly dependent on the size of the training data. In addition, the fingerprint data from different types of sensors may also influence algorithm performance^7^.

To overcome these issues, we developed a high-resolution, depth-resolving fingerprint imaging system based on photoacoustic (PA) tomography (PAT)^8^. PAT provides optical absorption contrast via the photoacoustic effect, which is the generation of ultrasound waves from light absorption. Due to weak ultrasound scattering in the tissue, PAT is able to overcome the optical diffusion limit and provide high-resolution images in deep tissue^9^. Fingerprint imaging using the photoacoustic effect is not entirely new. In vivo and latent fingerprint imaging were demonstrated in previous studies^10–12^. For instance, Choi et al*.* used a photoacoustic microscopy (PAM) system to image fingerprint of a dry finger. However, the study did not reveal any underlying vascular structures^12^. Song et al*.* demonstrated the imaging of latent fingerprints using an optical-resolution PAM system. While the system provides high-quality visualization of latent fingerprints, the authors did not demonstrate its capability in vivo^11^. More recently, Sun et al*.* captured the hierarchical vascular structure of finger using optical-resolution photoacoustic microscopy^10^. However, the field of view (FOV) was limited to 25 mm^2^ and the scanning time was about 250 s, which was too long for practical application. In this study, we developed a linear-array-based PAT system that is capable of providing high-resolution 3D images of fingerprints and the underlying vascular structures. Since both fingerprints and subdermal vasculature are acquired, PAT can counter-measure both bulk and thin-film fingerprint spoofs. Furthermore, we found that coupling through the acoustic gel is not required for this imaging system, which increases its potential for commercial translation. Our system can scan across the finger (FOV: 30 mm × 27.6 mm) within 60 s.

## Methods

### Imaging system construction

**Figure 1:**
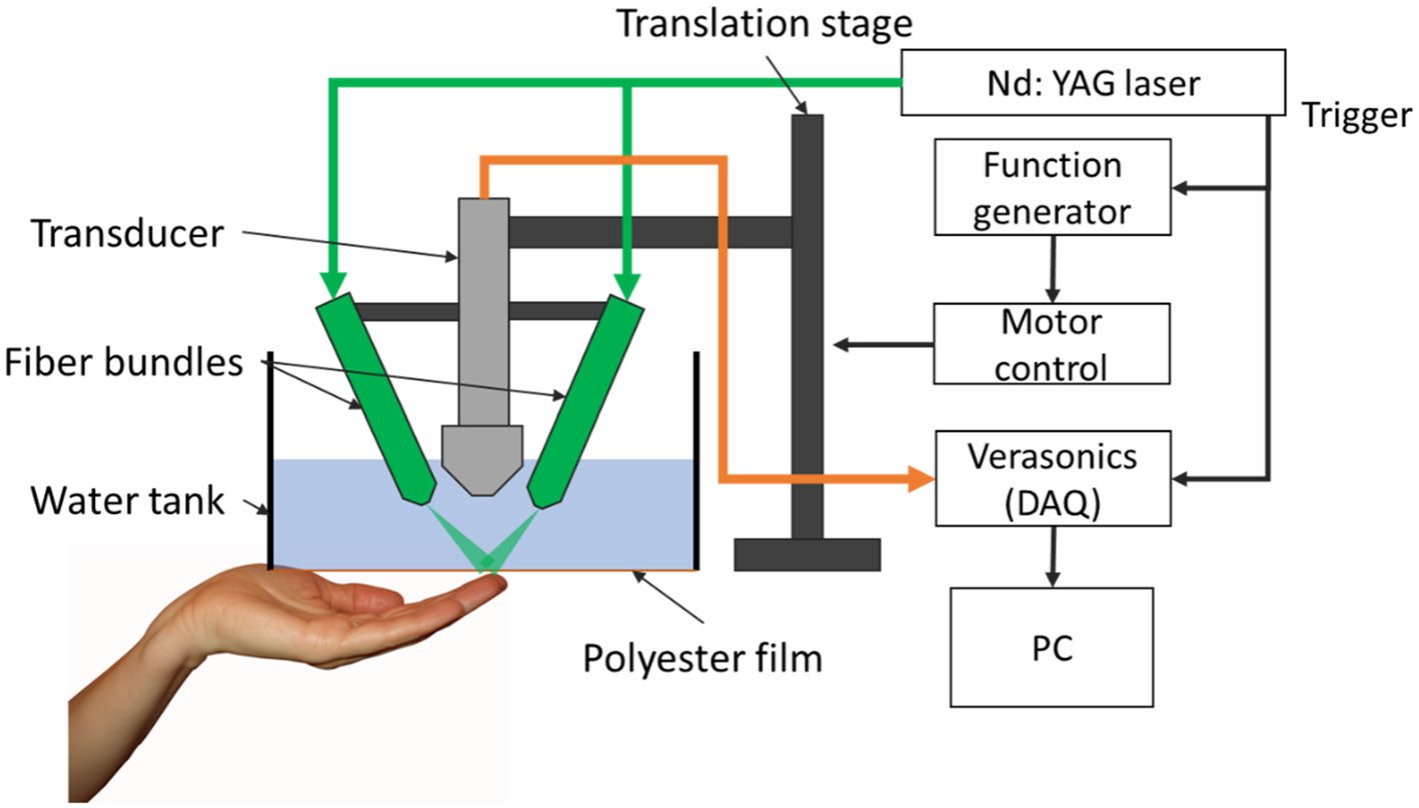
Schematic diagram of the PAT fingerprint imaging system.

Our PAT system consists mainly of three components: a 532-nm pulsed Nd: YAG laser with 10-Hz pulse repetition frequency (PRF) (Continuum, SL III), a 128-channel DAQ (Verasonics, Inc.), and a 256-element linear transducer array (L22-8, 15.6 MHz central frequency, Verasonics, Inc.) as shown in Fig. 1. The laser beam was first passed through the bifurcated fiber bundle with a 9-mm-diameter circular input and two 7.6 cm-long line outputs. Then, each line output was focused by a cylindrical lens to form line-shaped light beams. The two line-shaped fiber outputs were fixed on each side of the transducer using a lab-designed mount. The angles of the outputs were adjusted to ensure that the two linear light beams overlap at the transducer focal area. The laser fluence on skin surface was measured to be less than 7 mJ/cm^2^, which is below the safety limit of 20 mJ/cm^2^ at 532 nm^13^. The laser trigger output was used to synchronize the DAQ system. During imaging, the transducer was placed inside a lab-made water tank, which has an opening at the bottom sealed with a 0.001″-thick polyester film. The maximum pressure on film is roughly 1000 Pa while the water tank is full. The polyester film was rated to sustain much higher pressure. The subject’s hand was placed underneath the film and imaged through this window. The light and acoustic attenuations caused by the plastic film are negligible, according to our previous study^14^. A 20 cm translation stage (McMaster-Carr) was utilized for linear scanning of the ultrasound transducer. During the scanning, the finger surface was placed near the acoustic focus of the transducer (15 mm). Fingertips were pressed against the plastic film with or without gel coupling. Our system scanned the transducer over a 30-mm range, and the final field of view was 30 mm by 27.6 mm (lateral length of the transducer). The scanning time was 60 s at 0.05 mm per pulse step size. Because the Verasonics system requires two laser pulses to capture the signal from all 256 elements in the ultrasound transducer array, the actual step size between each imaging frame was 0.1 mm. For all experiments, the sampling rate was 62.5 MHz (4 × central frequency) and the amplification was 54 dB. All experimental protocols were approved by the University at Buffalo Institutional Review Board (IRB) and informed consent was obtained from all subjects (adults). The experimental procedures were performed in accordance with relevant guidelines and regulations. Informed consent for publication of the fingerprint images has been obtained from the study participant prior to this study.

### Data reconstruction and processing

Ultrasound B-mode images were reconstructed using the built-in function in the Verasonics system. Before image reconstruction, we used cross-correlation to align raw data among all the frames to eliminate DAQ jittering. Then, for PAT data, we first applied a band-pass filter (15–20 MHz) to remove the low-frequency noises in the signal. Next, we utilized the back-projection algorithm to reconstruct each 2D frame^15^. Then, we stacked all the frames along the scanning direction to form a 3D image. To better reveal the fingerprint structure and subdermal vasculature, we developed an algorithm to separate these two layers. The locations of these two layers were determined by averaging the signal intensities along lateral direction in each cross-sectional image. Therefore, signals from fingerprint and vessel layers can be separated. We also used the ultrasonic data as the ground truth and compared them with the PAT data.

## Results

### System performance quantification

**Figure 2:**
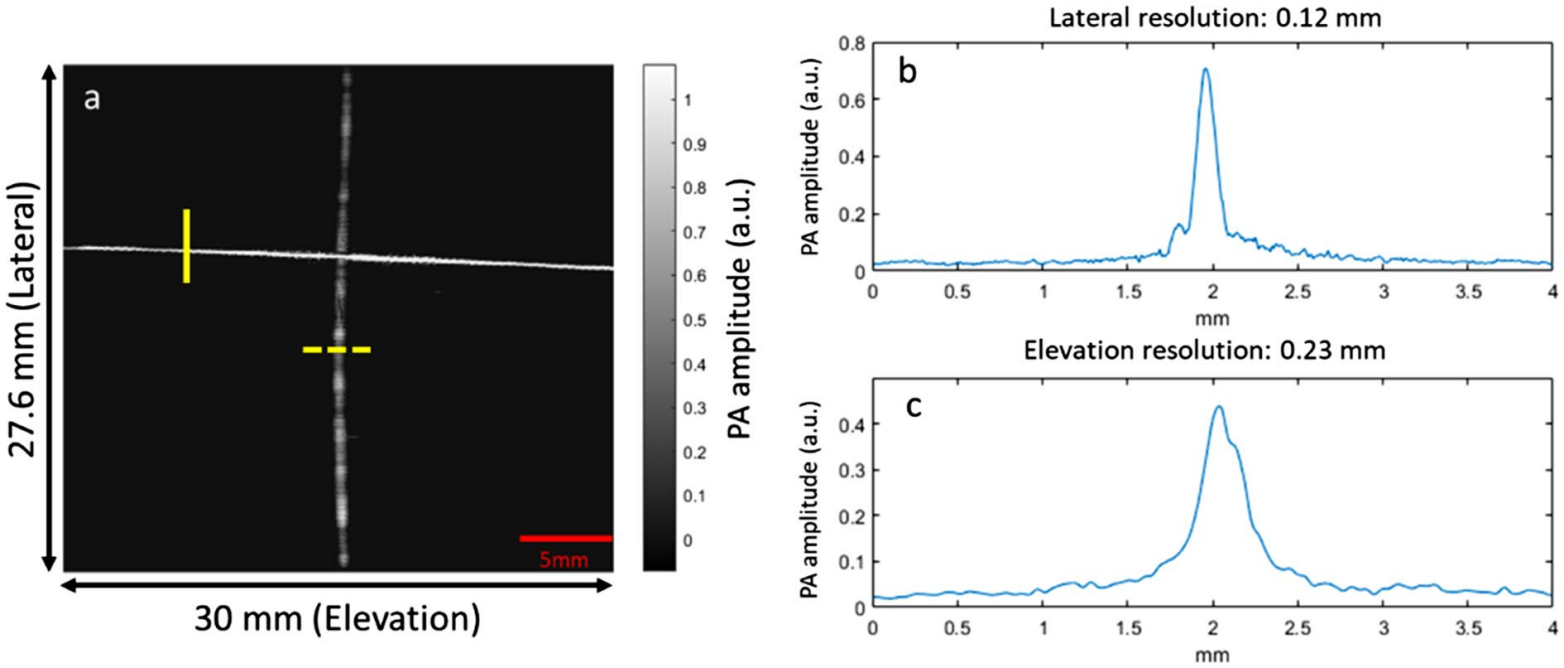
Quantification of lateral and elevation resolutions of the fingerprint imaging system, using human hair. (**a**) PAT image of two hairs perpendicular to each other. (**b**) Lateral resolution as quantified by PA signal intensity along the solid yellow line in (**a**). (**c**) Elevation resolution as quantified by PA signal intensity along the dashed yellow line in (**a**).

We first imaged human hair to quantify the spatial resolution of the system. As shown in Fig. 2a, two strands of 60 μm human hair were placed perpendicular to each other in the field of view of our imaging system. We quantified the full width at half maximum (FWHM) along lateral and elevation directions to be 0.12 mm (Fig. 2b) and 0.23 mm (Fig. 2c), respectively. The pitch of a typical fingerprint pattern is about 0.5 mm, which can be resolved by our system^3^. It should be noted that the lateral resolution could be slightly better than 0.12 mm if we use a smaller diameter wire, such as micrometer tungsten wires.

**Figure 3:**
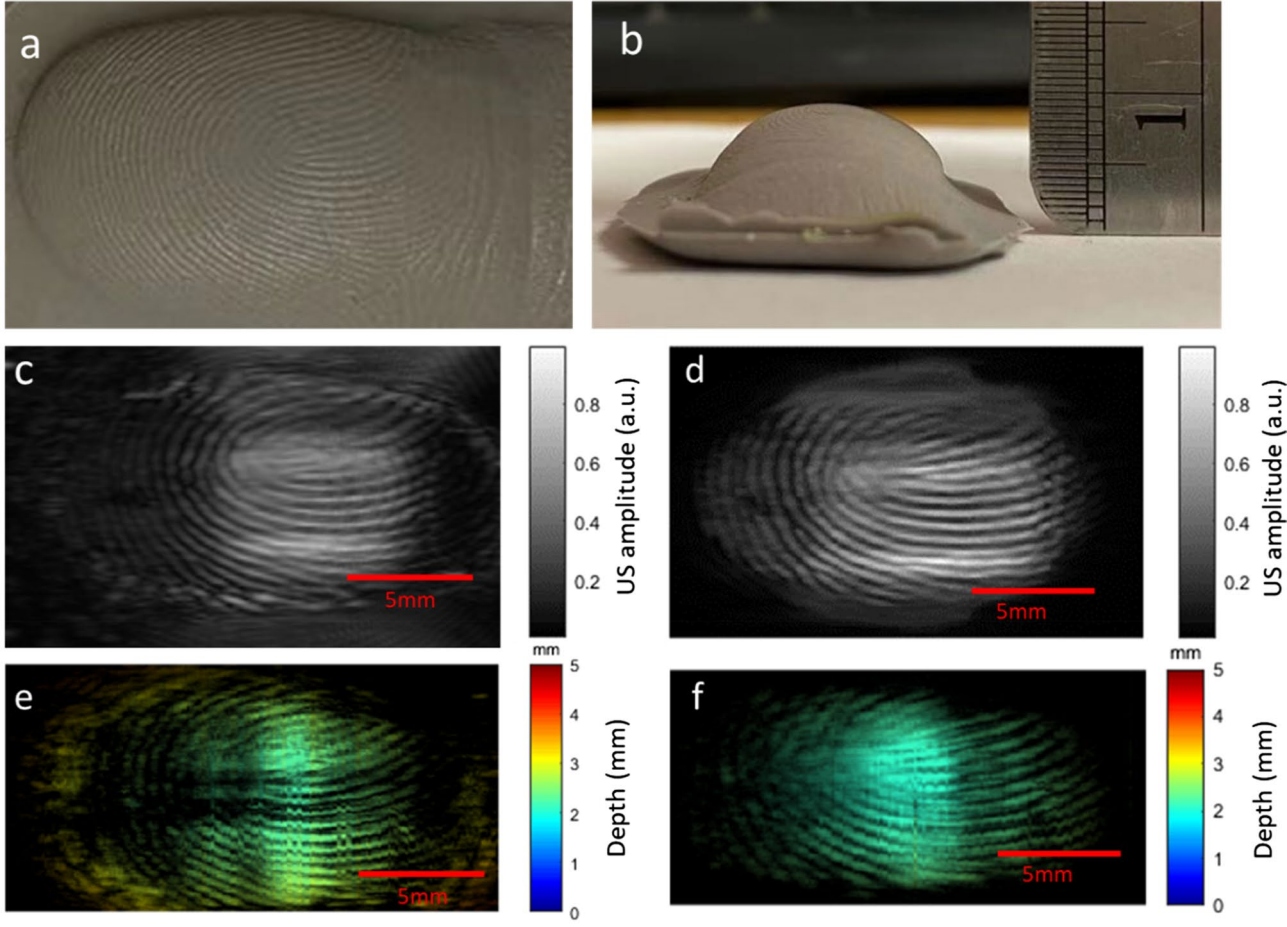
Imaging results for leaf phantom and artificial fingerprint spoof. (**a**) Photography of the top view of finger spoof. (**b**) Photography of the front view of finger spoof. (**c**) Ultrasound image of the spoof with coupling. (**d**) Ultrasound image of the spoof without coupling. (**e**) Depth-encoded photoacoustic image of the spoof with coupling. (**f**) Depth-encoded photoacoustic image of the spoof without coupling.

Next, we imaged a fingerprint spoof to further validate the system performance. The gray-colored spoof has strong light absorption at 532 nm (Fig. 3a,b). The ultrasound and photoacoustic images are shown in Fig. 3c,e, respectively. Fingerprint patterns can be observed in both images. To mimic the actual fingerprint scanning conditions, we also imaged the spoof without any ultrasound gel coupling. Figure 3d,f show the ultrasound and photoacoustic results, respectively. The contrast between the fingerprint ridges and valleys can still be observed in both images. These results indicate that the imaging system can effectively resolve features on the fingertip.

### In vivo imaging

**Figure 4:**
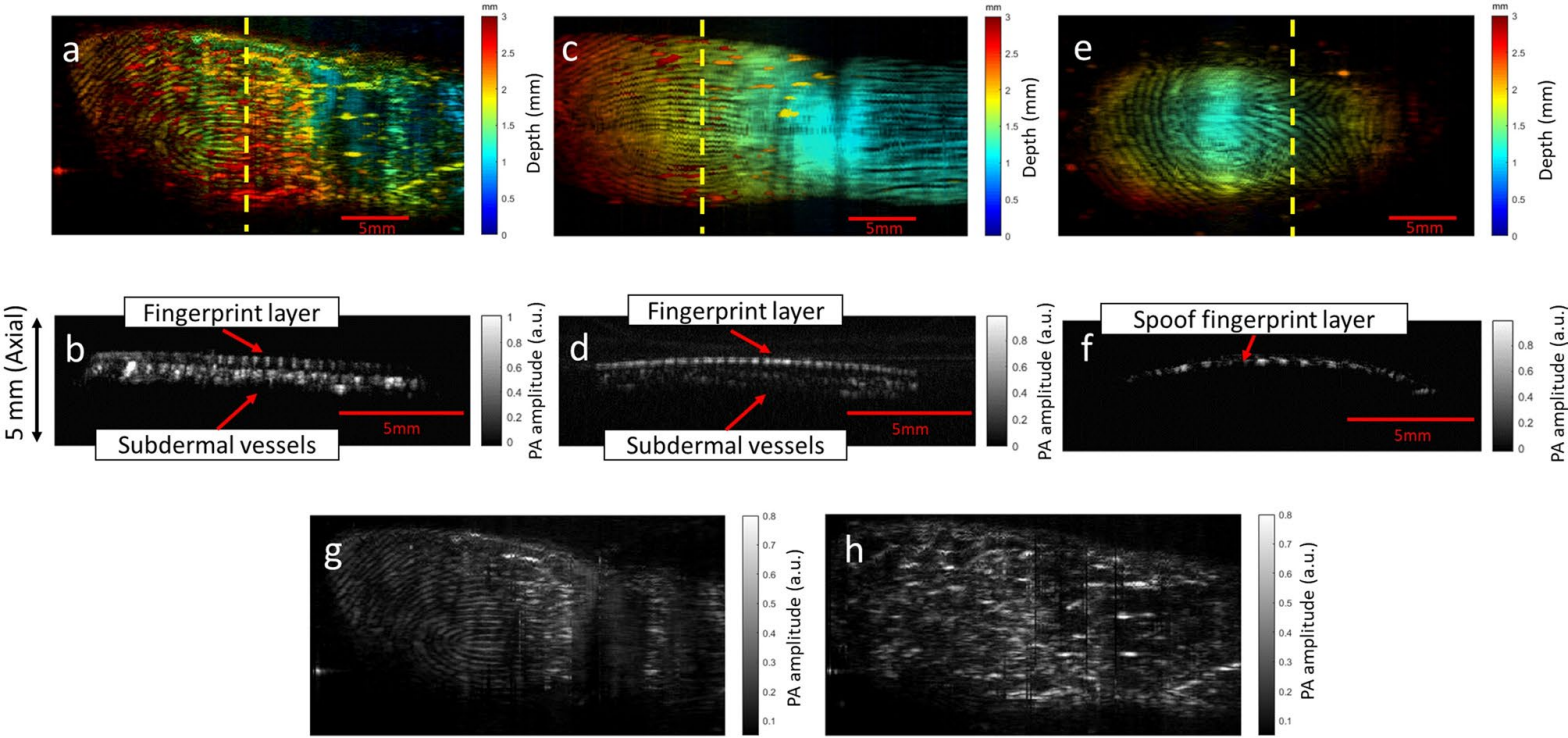
PAT results of human fingerprint. (**a**) Depth-encoded MAP image of the fingerprint obtained with ultrasound gel coupling. (**b**) Cross-sectional PAT image across the yellow dashed line in (**a**). (**c**) Depth-encoded MAP image of the fingerprint obtained without ultrasound gel coupling. (**d**) Cross-sectional PAT image across the yellow dashed line in (**c**). (**e**) Depth-encoded MAP image of the spoof fingerprint obtained with ultrasound gel coupling. (**f**) Cross-sectional PAT image across the yellow dashed line in (**e**). (**g**) Extracted fingerprint MAP image with ultrasound gel coupling. (**h**) Extracted subdermal vasculature MAP image with ultrasound gel coupling.

Following the spoof experiment, we imaged the human finger under different acoustic coupling conditions. First, we imaged the fingertip with ultrasound gel coupling. As shown in Fig. 4a, both the fingerprint and the subdermal vasculature can be observed in the depth-encoded image. The color scale from blue to red represents the depth ranges from 0 ~ 5 mm. Most of the fingerprint patterns are in green because they are closer to the transducer, while the vasculature, in red and yellow, lie underneath the fingerprint surface. A cross-sectional view of the result is shown in Fig. 4b. Fingerprint patterns can be clearly observed at the top layer and the fingerprint pitch between ridges was measured at around 0.4 mm, which agrees with the typical values mentioned in Ref.^3^. Additionally, the subdermal vasculatures underneath the surface layer are also visible at the second layer of Fig. 4b. Results obtained without ultrasound gel coupling are shown in Fig. 4c,d. These images exhibit more defined fingerprint structures but less subdermal vascular information. These results are expected because the dry finger increases acoustic impedance mismatch between the finger and coupling membrane. As will be seen in the discussion section, the vascular structures can be enhanced through depth weighting. The spoof fingerprint image is shown in Fig. 4e. The cross-sectional image in Fig. 4f did not show any vascular patterns or tissue layers. Figure 4g,h show the extracted fingerprint image and subdermal vasculature with ultrasound gel coupling, respectively. It can be seen that most of the vessel structures were separated from the fingerprint, except the middle top portion, as the vessels in that region are too close to the fingerprint layer.

**Figure 5:**
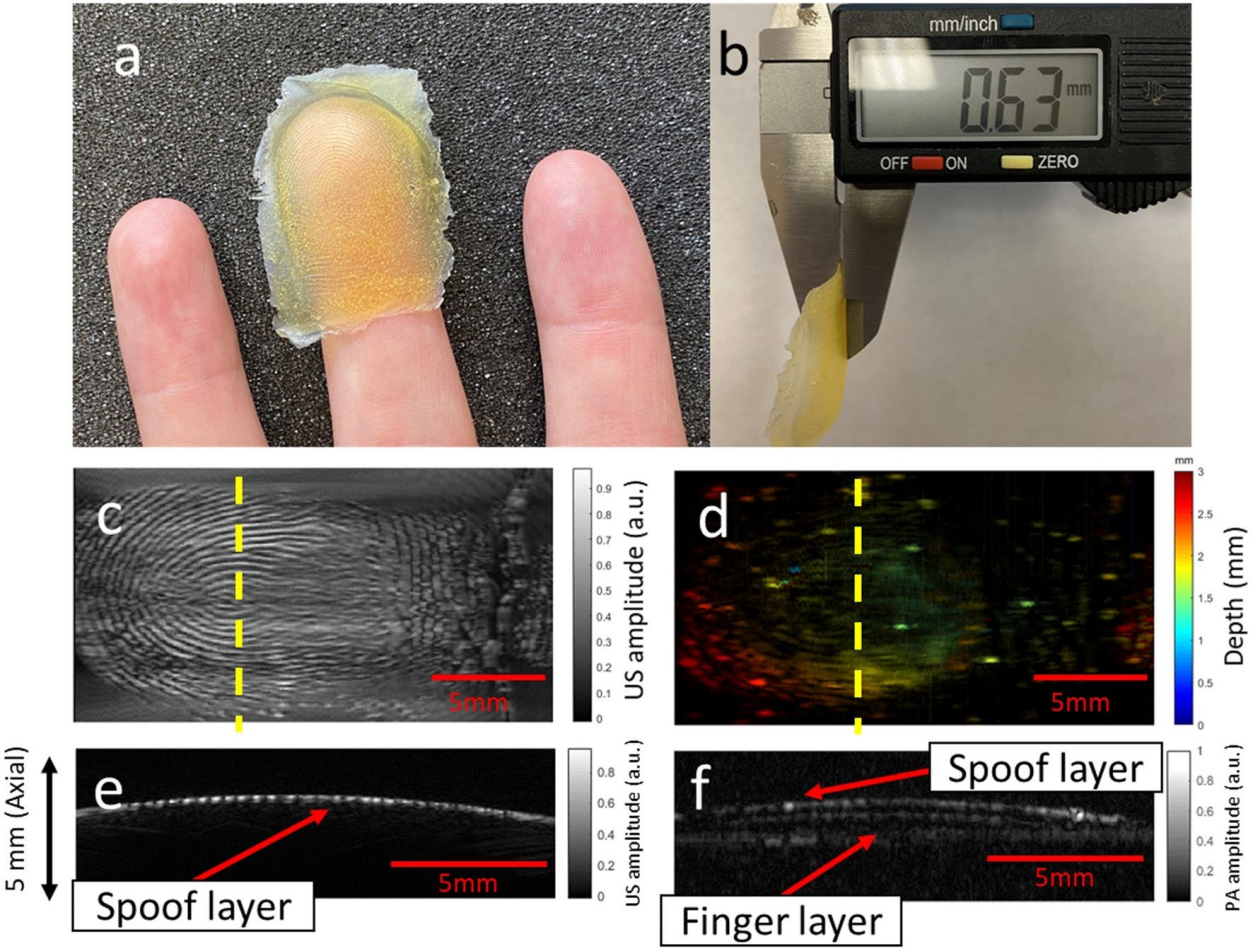
Imaging results of the thin-film spoof. (**a**) Photograph of spoof placed on human finger. (**b**) Thickness-measurement of spoof. (**c**) Ultrasound image. (**d**) Photoacoustic image. (**e**) Cross-sectional ultrasound image across the yellow dashed line in (**c**). (**f**) Cross-sectional PAT image across the yellow dashed line in (**d**).

Thin-film artificial fingerprints represent another common hacking technique^16^. Thin-film spoofs are typically applied to the surface of a real finger. To investigate the performance of PAT on thin-film spoofs, we conducted an experiment with a 0.63-mm-thick fingerprint spoof formed from wood glue (Fig. 5a,b). The spoof fingerprint patterns were made from another subject’s fingerprints. The ultrasound and PAT images are shown in Fig. 5c,d, respectively. The ultrasound image clearly shows the spoof fingerprint, while the PAT-imaged fingerprint is unclear due to the translucence color of the spoof. This is an advantage for PAT because we do not want the spoof to show strong fingerprint contrast. In the cross-sectional images, the ultrasound result (Fig. 5e) shows a single layer, while the PAT result (Fig. 5f) clearly indicates the presence of two fingerprint layers and the underlying vascular features. This result is expected because ultrasound relies on round-trip acoustic propagation and most of the acoustic energy was reflected by the first layer. PAT is based on one-way acoustic propagation, and therefore deeper layers can be visualized.

**Figure 6:**
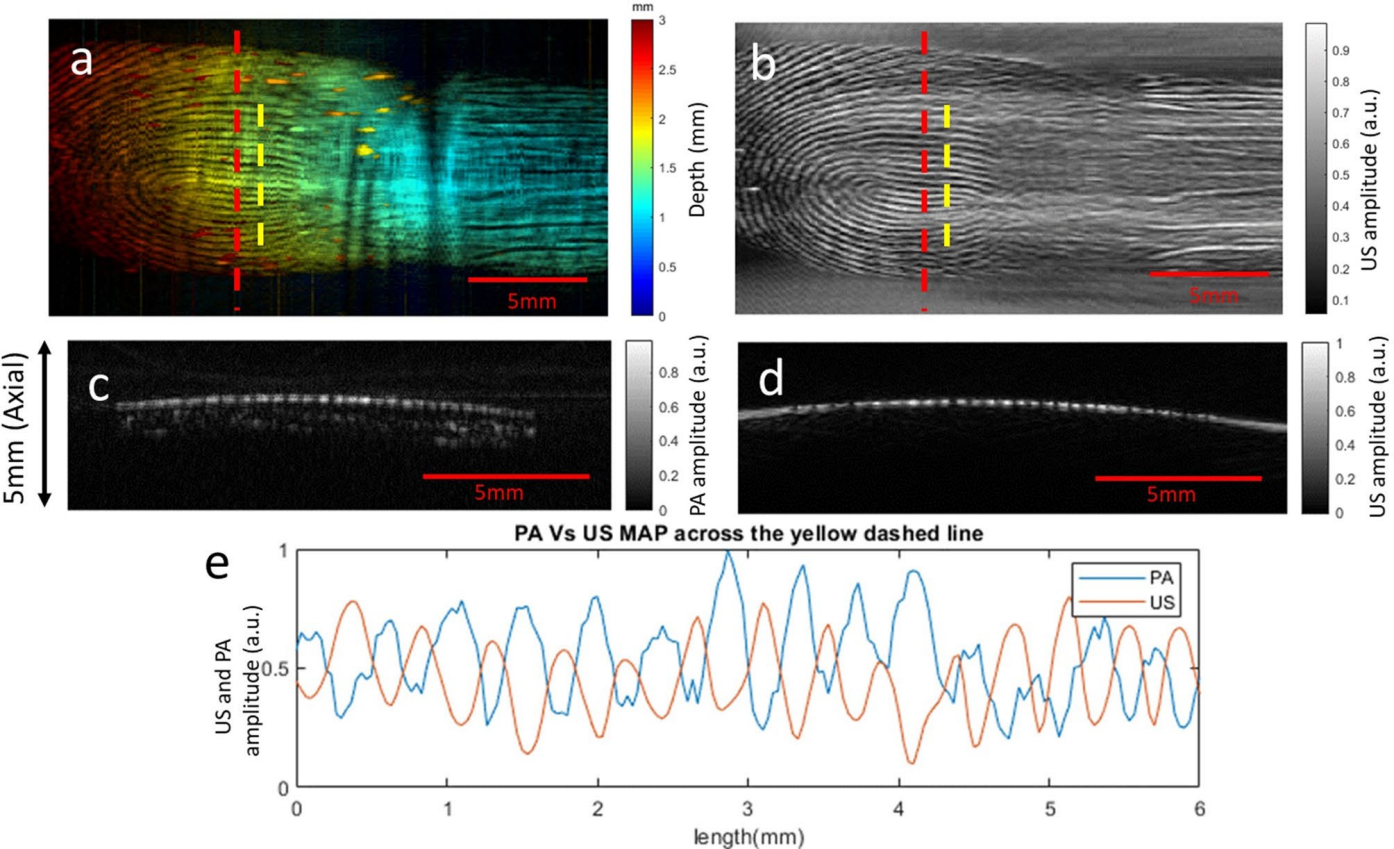
Photoacoustic and ultrasound images of a dry finger. (**a**) Depth-encoded photoacoustic image. (**b**) Gray-scale ultrasound image. (**c**) Cross-sectional PAT image across the red dashed line in (**a**). (**d**) Cross-sectional US image across the red dashed line in (**c**). (**e**) Intensity profiles of the ultrasound and photoacoustic images across the yellow dashed lines in both (**a**) and (**b**).

We also compared the contrasts originating from photoacoustic (Fig. 6a) and ultrasound (Fig. 6b) fingerprint images of dry fingers. The cross-sectional PAT and US image across the red dashed line are shown in Fig. 6c,d, respectively. For the PA image, we removed the subdermal layer to clearly reveal the fingerprint structure. The signal profiles along the yellow dashed line are presented in Fig. 6e. It can be seen that valleys generate stronger signals in ultrasound images, while ridges exhibit higher signal amplitude in PA images. This is because ultrasound fingerprint imaging relies on the acoustic impedance mismatch between the fingerprint valley and plastic membrane. The mismatch generates strong acoustic echoes. For PAT, the mismatch reduces the propagation of PA signals from the fingerprint valley to the transducer. As can be seen in Fig. 6e, the ridge and valley contrasts in PAT and ultrasound are clearly inverted.

## Discussion

We have developed a PAT system that can reveal both fingerprint and subdermal vascular structures. We found that hemoglobin in the fingerprint layer and subdermal vasculature is the absorbing component for PA signal generation. The impedance mismatch provides the contrast for fingerprint. The vascular features can be used for either liveness verification or as an additional biometric feature for identification^17,18^. Therefore, The 3D PAT images provide more biometric features compared to other fingerprint imaging modalities. As mentioned above, our system can serve as a counter-measure against artificial fingerprints by utilizing the information from different depths. Even if the artificial fingerprint is a thin film that contains high optical absorption to block the real fingerprint layer. Our system can still identify it as the underlying vessel would be missing, which is a key feature of liveness detection. Moreover, our system simultaneously acquires PA and pulse-echo ultrasound images. The ultrasonic fingerprint features can be utilized for PA fingerprint imaging validation.

However, as discussed in the previous section, less vasculature information was obtained from the dry coupling experiment, due to the impedance mismatch caused by air gaps at fingerprint valleys. This issue can be addressed through post-processing. Since lower frequency signals contain greater structural information, we filtered the raw channel data to 5 ~ 8 MHz to remove the fingerprint features. After image reconstruction, we applied a depth-enhanced weighting function to increase signals from the subdermal layer. The equation of the weighting function is shown below.

1 $${\text{f}}\left( {x_{n} } \right) = \frac{a}{{1 + e^{{ - b*x_{n} }} }}.$$

**Figure 7:**
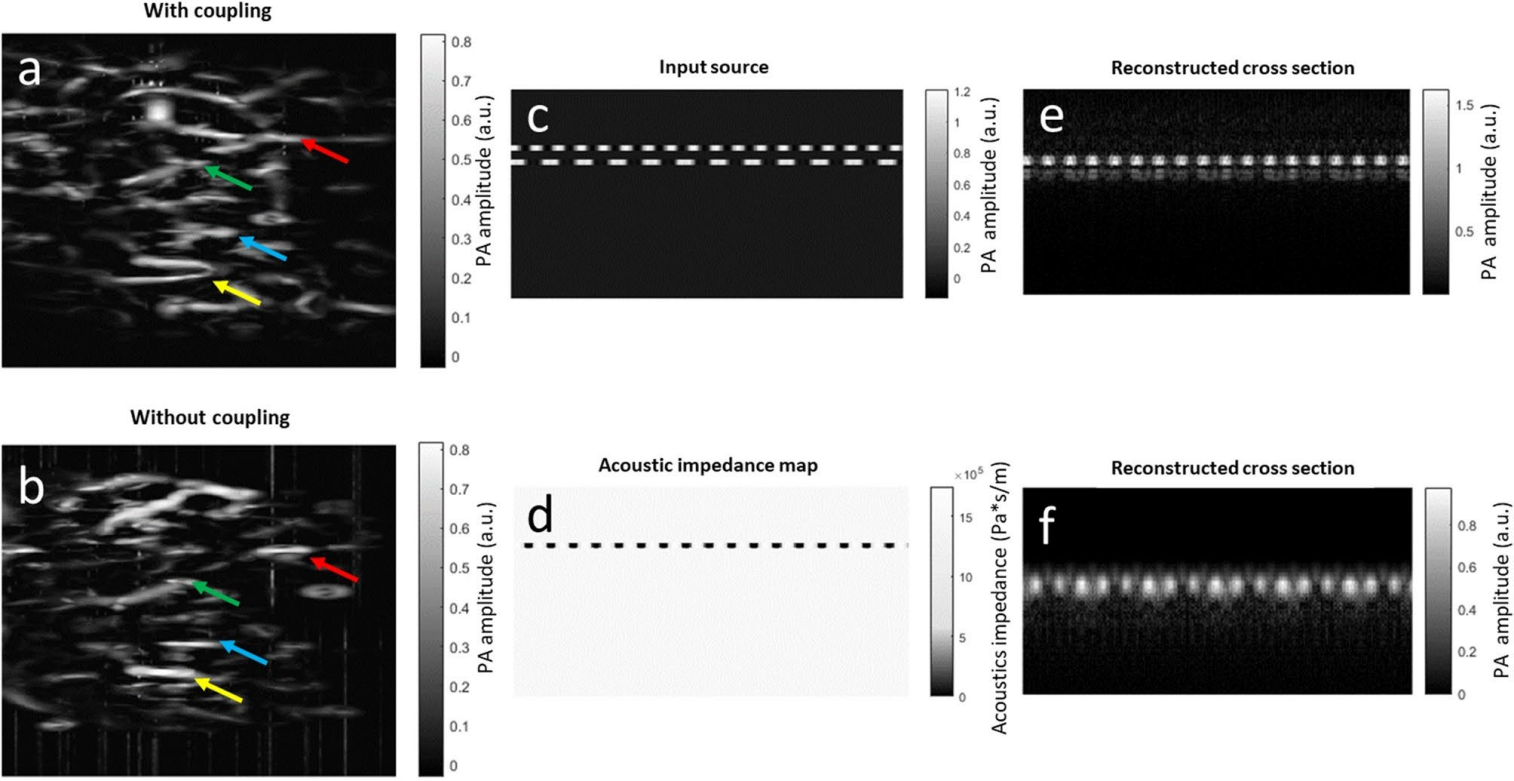
Extraction of vascular structure in dry coupling. (**a**) Vessel-enhanced MAP image of the fingertip with gel coupling. (**b**) Vessel-enhanced MAP image of the fingertip witout gel coupling. (**c**) Distribution of the optical absorber defined in the K-wave simulation environment. (**d**) Distribution of acoustic impedance defined in the K-wave simulation environment. White represents water and black represents air. (**e**) The original reconstructed cross-sectional image under the simulation conditions in (**c**) and (**d**). (**f**) Enhanced image of the second layer.

Here,${x}_{n}$ is the pixel intensity of numerical index $n$ of the A-line. Constant *a* determines the maximum value of the curve, and constant *b* modulates the steepness of the curve to match the position of the underlying vessels. Then, we applied the Frangi filter^19^ to further extract the vessel-like structures. We also employed this processing on fingerprint image in gel coupling conditions. The results shown in Fig. 7a,b indicate that fingerprint structures were removed by the low-frequency bandpass filter, and the subdermal vessels were enhanced by the weighting function and vessel filter. The two vessel-enhanced images demonstrate similar vessel features. The common structures are indicated by arrows with the same colors in both images. It can be noticed that most vessels extend along the horizontal direction. This observation agrees with literature^20^, which states that most branching arteries in the fingertip extend transversely.

To validate the vessel-enhanced approach, we conducted MATLAB-based simulations using the K-wave toolbox and applied the vessel-enhanced method to the simulated data^21^. An input source was created based on the cross-sectional image of the ridges and the valleys on the fingertip. As shown in Fig. 7c, the bright spots represent higher optical absorption. The top and bottom layers mimic the fingerprint and subdermal vessel layers, respectively. The thicknesses of both layers were defined based on the experimental data. In the first layer, the medium properties between two optical absorbers were defined as air to mimic the valley of the fingerprint. Besides fingerprint valleys, all other regions have the same acoustic property of water, as shown in Fig. 7d. The receiver parameters were defined to be similar to that of the experiment. The reconstructed cross-sectional image of simulated data is shown in Fig. 7e. The fingerprint layer is clearly revealed in the reconstructed image. Minor broadening in the fingerprint features was observed, which is possibly due to the relatively low spatial resolution of the transducer. The PA intensities of the first layer were much stronger than that of the second layer due to impedance mismatch at the fingerprint valley. We then applied the vessel-enhanced approach to the data of dry finger simulation. The first layer was removed and the second layer can be clearly observed (Fig. 7f).

While promising results have been acquired, this proof-of-concept study has some limitations. For instance, our system’s spatial resolution is still not as good as photoacoustic microscopy (typically 84 μm^22^). While the lateral resolution of our system is better than most array-based PAT systems, the linear array used in our study has intrinsic limitations along the elevation direction (resolution along the scanning direction). The spatial resolution can be increased by using a higher frequency linear array or a matrix array, or utilizing advanced 3D image reconstruction algorithms^14,23^. Moreover, using polyester film for finger coupling is not practical in system commercialization. It can be seen that the arch fingerprint pattern of the spoof was not fully revealed because the polyester film is not stiff enough to deform the spoof. Therefore, the arch pattern in this spoof cannot be revealed properly. However, the arch pattern of the human finger in the in vivo imaging can be clearly revealed as the human finger was deformed and entirely attached to the polyester film, which are shown in Fig. 4a,c. Clear glass or plastic sheets can be used in the future. These materials will likely enhance the fingerprint feature due to larger acoustic mismatch and will slightly reduce the subdermal PA vascular signals. Another limitation is the imaging speed. The current imaging time is 60 s, which is slower than commercial ultrasonic fingerprint scanners built on a 2D matrix array. Our system’s imaging speed is mainly limited by the 10-Hz pulsed laser. By using a higher frequency laser (e.g., 1 kHz laser diode array from Quantel Laser), we will be able to improve the scanning time to less than 10 s^24,25^. In addition, while signal alignment was employed to calibrate the jittering of DAQ, some ripples still exist. In future studies, we can completely eliminate jittering by using DAQ to trigger laser firing. Moreover, the current system is relatively bulky and difficult to be used in commercial settings. To reduce the system size, we can utilize a 2D ultrasonic sensor combined with a compact DAQ^26,27^. The snapshot photoacoustic topography system could also be a potential solution as it relies on the ergodic relay principle to reduce the system size and complicity significantly^28,29^. Nevertheless, this proof-of-concept study demonstrated the potential of PAT for improved fingerprint imaging and we expect that future improvements will eventually move the technique closer to commercialization.

## Conclusion

In this study, we developed a linear ultrasound array-based fingerprint imaging system that can image fingerprints based on both ultrasound and photoacoustic contrasts. Our result indicates that PAT can be utilized in fingerprint imaging and its signal contrast is inverted to that of ultrasound. Combining the two contrasts will allow the visualization of both fingerprint valleys and ridges. Moreover, PAT can reveal dermal and subdermal vascular structures, which could be used for liveness detection or as a secondary identification feature^18^. While PAT typically requires ultrasound gel coupling, our study indicates that the system also works efficiently on the dry finger where the fingerprint structure can be observed by acoustic impedance mismatch. We also enhanced the attenuated sub-fingerprint features by employing frequency filtering and depth-dependent weighting. We envision that PAT can be combined with ultrasound in the future to bring fingerprint imaging to the next level, with enhanced anti-spoof and liveness detection capabilities as well as intrinsic finger vessel sensing.
